# Multicopy Single-Stranded DNA Directs Intestinal Colonization of Enteric Pathogens

**DOI:** 10.1371/journal.pgen.1005472

**Published:** 2015-09-14

**Authors:** Johanna R. Elfenbein, Leigh A. Knodler, Ernesto S. Nakayasu, Charles Ansong, Heather M. Brewer, Lydia Bogomolnaya, L. Garry Adams, Michael McClelland, Joshua N. Adkins, Helene L. Andrews-Polymenis

**Affiliations:** 1 Department of Microbial Pathogenesis and Immunology, College of Medicine, Texas A&M University Health Science Center, Bryan, Texas, United States of America; 2 Department of Clinical Sciences, College of Veterinary Medicine, North Carolina State University, Raleigh, North Carolina, United States of America; 3 Paul G. Allen School of Global Animal Health, College of Veterinary Medicine, Washington State University, Pullman, Washington, United States of America; 4 Integrative Omics Group, Biological Sciences Division, Pacific Northwest National Laboratory, Richland, Washington, United States of America; 5 Environmental Molecular Sciences Laboratory, Pacific Northwest National Laboratory, Richland, Washington, United States of America; 6 Department of Veterinary Pathobiology, College of Veterinary Medicine, Texas A&M University, College Station, Texas, United States of America; 7 Department of Pathology and Laboratory Medicine, School of Medicine, University of California-Irvine, Irvine, California, United States of America; University of Washington, UNITED STATES

## Abstract

Multicopy single-stranded DNAs (msDNAs) are hybrid RNA-DNA molecules encoded on retroelements called retrons and produced by the action of retron reverse transcriptases. Retrons are widespread in bacteria but the natural function of msDNA has remained elusive despite 30 years of study. The major roadblock to elucidation of the function of these unique molecules has been the lack of any identifiable phenotypes for mutants unable to make msDNA. We report that msDNA of the zoonotic pathogen *Salmonella* Typhimurium is necessary for colonization of the intestine. Similarly, we observed a defect in intestinal persistence in an enteropathogenic *E*. *coli* mutant lacking its retron reverse transcriptase. Under anaerobic conditions in the absence of msDNA, proteins of central anaerobic metabolism needed for *Salmonella* colonization of the intestine are dysregulated. We show that the msDNA-deficient mutant can utilize nitrate, but not other alternate electron acceptors in anaerobic conditions. Consistent with the availability of nitrate in the inflamed gut, a neutrophilic inflammatory response partially rescued the ability of a mutant lacking msDNA to colonize the intestine. These findings together indicate that the mechanistic basis of msDNA function during *Salmonella* colonization of the intestine is proper production of proteins needed for anaerobic metabolism. We further conclude that a natural function of msDNA is to regulate protein abundance, the first attributable function for any msDNA. Our data provide novel insight into the function of this mysterious molecule that likely represents a new class of regulatory molecules.

## Author Summary

Multicopy single-stranded DNA (msDNA) is a unique molecule consisting of both an RNA and DNA portion. This molecule is produced by a reverse transcriptase and has no known natural function despite more than 30 years of study. We report that msDNA is important for both *Salmonella* Typhimurium and an enteropathogenic *E*. *coli*, two pathogens that cause diarrhea in susceptible hosts, to survive in the intestine. Using mutant strains incapable of producing msDNA, we show that msDNA is needed for *Salmonella* to grow in the absence of oxygen. Mutants grown in oxygen-deficient conditions have substantial changes in overall protein composition, including numerous proteins known to be important for anaerobic metabolism and growth in the intestine. Our findings link msDNA to the ability of *Salmonella* to thrive in an oxygen-deficient environment similar to the conditions inside the gut. We report that msDNA regulates the quantity of proteins, the first natural function attributed to this molecule. msDNA may represent a new class of regulatory molecules.

## Introduction

Retron reverse transcriptases (RT) in bacteria were first described in *Myxococcus xanthus* [1] and *E*. *coli* [2] in the 1980s and are now known to be widely distributed in the genomes of eubacteria and archaea (reviewed in [3]). All retrons contain three regions essential for production of msDNA: *msr* (RNA primer for reverse transcription), *msd* (template sequence), and a reverse transcriptase (RT). The retrons of pathogens, such as *Salmonella* Typhimurium (STm), may also encode an additional ORF of unknown function [4]. The product of the ‘retron’ is a small covalently linked RNA-DNA hybrid molecule called multicopy single-stranded DNA (msDNA) that is predicted to form complex secondary structures [5]. The predicted secondary structures of msDNA from enteric pathogens including STm, enteropathogenic *E*. *coli* and *Vibrio spp*. are similar [4] but the reverse transcriptase amino acid sequence from these enteric pathogens share little identity. The location of the retron as well as the number of retrons in each species varies. These observations suggest that retrons have been horizontally acquired by convergent evolution to function in a fashion that is specific to the biology of the host bacterium.

Although the molecular details of the production of msDNA have been heavily studied, no natural function has been attributed to this mysterious molecule despite 30 years of study (reviewed in [3]). A critical obstacle to elucidating the natural function of msDNA was the lack of any phenotype for mutants unable to make this molecule. We have shown that the retron reverse transcriptase encoded by *STM3846* is essential for *Salmonella* Typhimurium (STm) to colonize the calf intestine [6], a natural model of enteric salmonellosis that recapitulates the earliest stages of human non-typhoidal *Salmonella* (NTS) infection. This was the first reported phenotype for a mutant lacking a retron reverse transcriptase.

NTS are major threats to global animal and human health, causing more than 90 million cases of gastroenteritis in people worldwide [7]. Human enteric salmonellosis is characterized by inflammatory diarrhea containing primarily neutrophils. To efficiently colonize the host, NTS use the type 3-secretion system 1 (T3SS-1) encoded on *Salmonella* Pathogenicity Island-1 (SPI-1) to invade the intestinal epithelium [8,9] and to promote the characteristic neutrophilic inflammatory response. The host inflammatory response gives *Salmonella* a competitive advantage over resident microflora. Within the intestinal lumen, the product of the neutrophilic oxidative burst generates tetrathionate from oxidation of thiosulfate [10]. *Salmonella* uses tetrathionate as a terminal electron acceptor within the anaerobic conditions of the intestinal lumen to gain a competitive advantage over resident microflora. Effectors of the TTSS-1 may directly activate epithelial production of inducible nitric oxide synthase (iNOS) thereby creating nitrate, an additional terminal electron acceptor [11]. The relative importance of nitrate during infection is illustrated by the fact that it is a powerful chemoattractant for *Salmonella* during anaerobiosis [12]. In addition, *Salmonella* uses host-derived nutrients such as ethanolamine [13] during intestinal inflammation. These strategies facilitate the growth of *Salmonella* in the complex microbial community of the intestine.

We used the enteric pathogen, *Salmonella* Typhimurium, to dissect the function of msDNA. In the work described here, we report that mutants lacking msDNA produced by the *STM3846* reverse transcriptase are defective for colonization of the intestine using murine models of salmonellosis. This colonization defect is due, in part, to a growth defect for these mutants in anaerobic conditions. We show that mutants lacking msDNA have altered abundance of over 200 proteins in anaerobiosis, many of which are known to be required for growth in anaerobic conditions and for the pathogenesis of STm during enteric infection. Inappropriate abundance of proteins encoding alternate terminal electron acceptor reductases results in an inability of mutants lacking msDNA to utilize these compounds, inhibiting anaerobic growth *in vitro*. The mutants lacking msDNA can only utilize nitrate as an anaerobic terminal electron acceptor. Mutants lacking msDNA fail to colonize portions of the intestine lacking substantial neutrophilic inflammation, likely due to the ability to only utilize nitrate to support anaerobic growth. Finally, we report a similar defect in intestinal persistence for an enteropathogenic *E*. *coli* lacking its retron reverse transcriptase suggesting that msDNA is critical for enteric pathogens to thrive in the intestine of mammalian hosts. Thus, we report a role in regulating protein abundance for msDNA, the first reported natural function for any msDNA. msDNA may represent a new class of bacterial regulatory molecules.

## Results

### msDNA is critical for STm intestinal colonization

**Fig 1:**
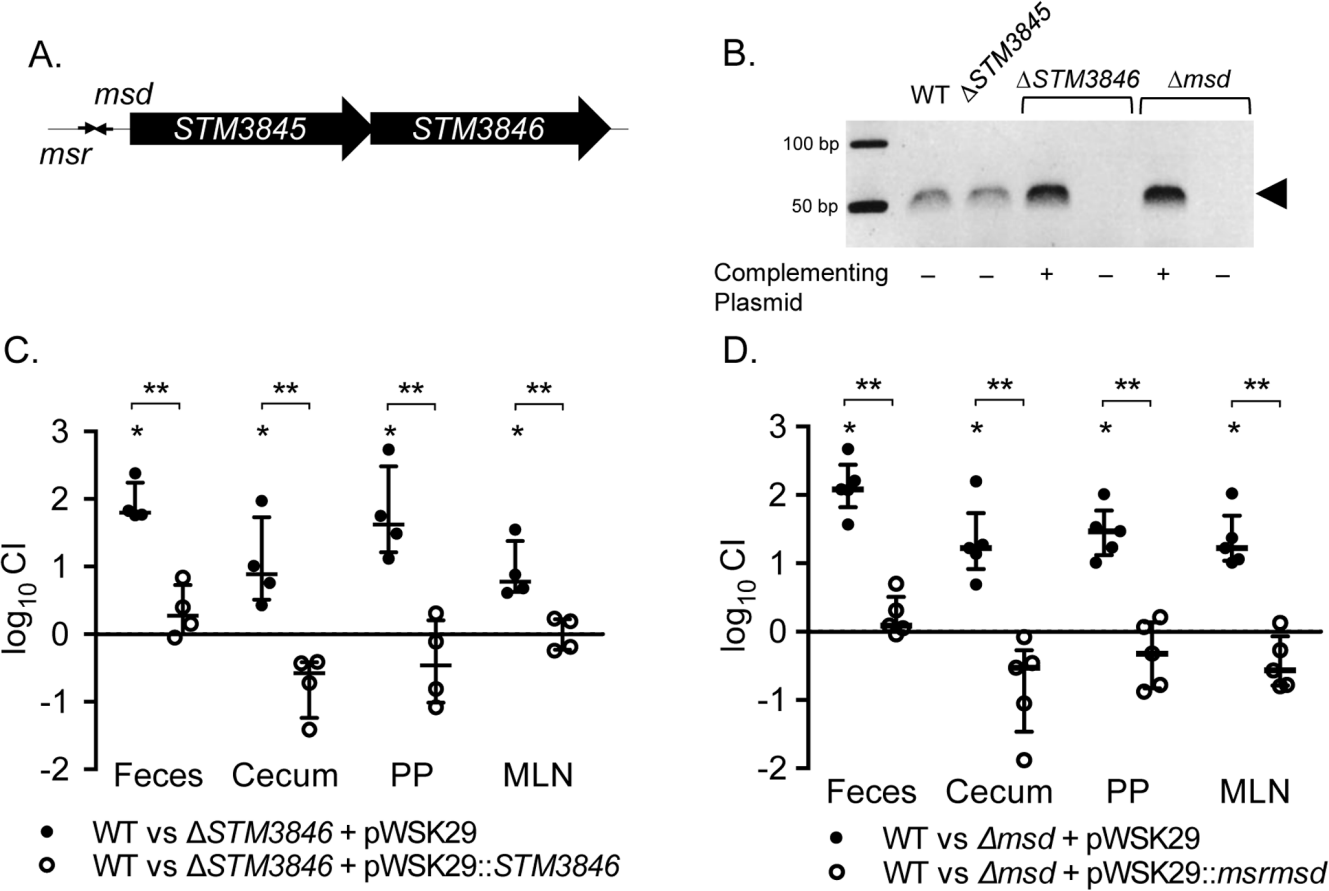
msDNA is produced by *msd* and STM3846 and is essential for *Salmonella* to colonize the intestine. (A) Genomic context of the retron St-85 (adapted from [6]). (B) msDNA was isolated from late-exponential phase cultures normalized by OD600 and visualized on a native 12% polyacrylamide gel with in-gel ethidium bromide staining. msDNA is depicted by the arrowhead (WT—HA420; Δ*STM3845*—JE143; Δ*STM3846* + pWSK29—JE122; Δ*STM3846* + p*STM3846*—HA1446; Δ*msd* + pWSK29—JE144; Δ*msd* + pRetronpro—JE145). (C-D) C57Bl/6 mice were treated orally with 20 mg streptomycin and infected with an equivalent mixture of (C) WT (HA697) and Δ*STM3846* bearing empty vector (closed circles; n = 4; JE63) or WT and complemented Δ*STM3846* mutant (open circles; n = 4; JE65) 24-hours after antibiotic treatment or (D) WT and Δ*msd* bearing empty vector (closed circles; n = 5; JE156) or WT and complemented Δ*msd* mutant (open circles; n = 5; JE154). Feces were collected 24-hours after infection and mice were euthanized 96-hours post-infection to harvest cecum, Peyer’s patches (PP), and mesenteric lymph nodes (MLN). Organ homogenates were serially diluted and plated to enumerate CFU. Competitive index (CI) was determined by comparing the ratio of WT to mutant in the organ with that of the inoculum. Each data point represents data from a single animal with median and interquartile range indicated. Statistical significance was determined by a t-test with * P < 0.05 (WT vs mutant) and ** P < 0.05 (between infection groups).

Retron reverse transcriptases, including the *STM3846* reverse transcriptase of the St-85 retron, use *msr* to prime reverse transcription of the *msd* template sequence to produce msDNA [14] (Fig 1A). We generated a non-polar deletion of *msd* to establish that msDNA, and not some other potential product of the STM3846 RT, mediates STm colonization of the intestine. Neither the Δ*STM3846* mutant nor the Δ*msd* mutant produce msDNA and its production can be restored in both mutants by complementation *in trans* (Fig 1B). The additional ORF, *STM3845*, is dispensable for msDNA production.

We used the murine colitis model [15], which responds to NTS infection with profound neutrophilic inflammation in the cecum, to dissect the function of the retron in intestinal colonization. We confirmed the requirement for *STM3846* in colonization of the inflamed intestine in this model (Fig 1C). In addition, both the Δ*msd* and Δ*STM3846* mutants have indistinguishable phenotypes, suggesting that the effect of deletion of the RT is mediated by the msDNA itself. The ability of each of these mutants to colonize the intestine is rescued by complementation *in trans* (Fig 1C and 1D). In cell culture, only the Δ*msd* mutant invades epithelial cells at a level mildly reduced compared to the isogenic wild type (S1 Fig) suggesting that reduced tissue invasion is unlikely to be the cause of the phenotype that we observed during infection of animal models. Our findings definitively link msDNA to the ability of *Salmonella* to colonize the intestine.

### msDNA and anaerobiosis

The intestine is a specialized and highly diverse niche. Oxygen tensions within the lumen decline from the stomach to the colon [16,17], and there is a gradient of increasing oxygen tension from the center of the lumen towards the epithelium [18]. Enteric pathogens must replicate in this hypoxic setting using both aerobic and anaerobic metabolic pathways [19,20] and express genes necessary for virulence in order to compete with resident microflora and colonize the host efficiently.

**Fig 2:**
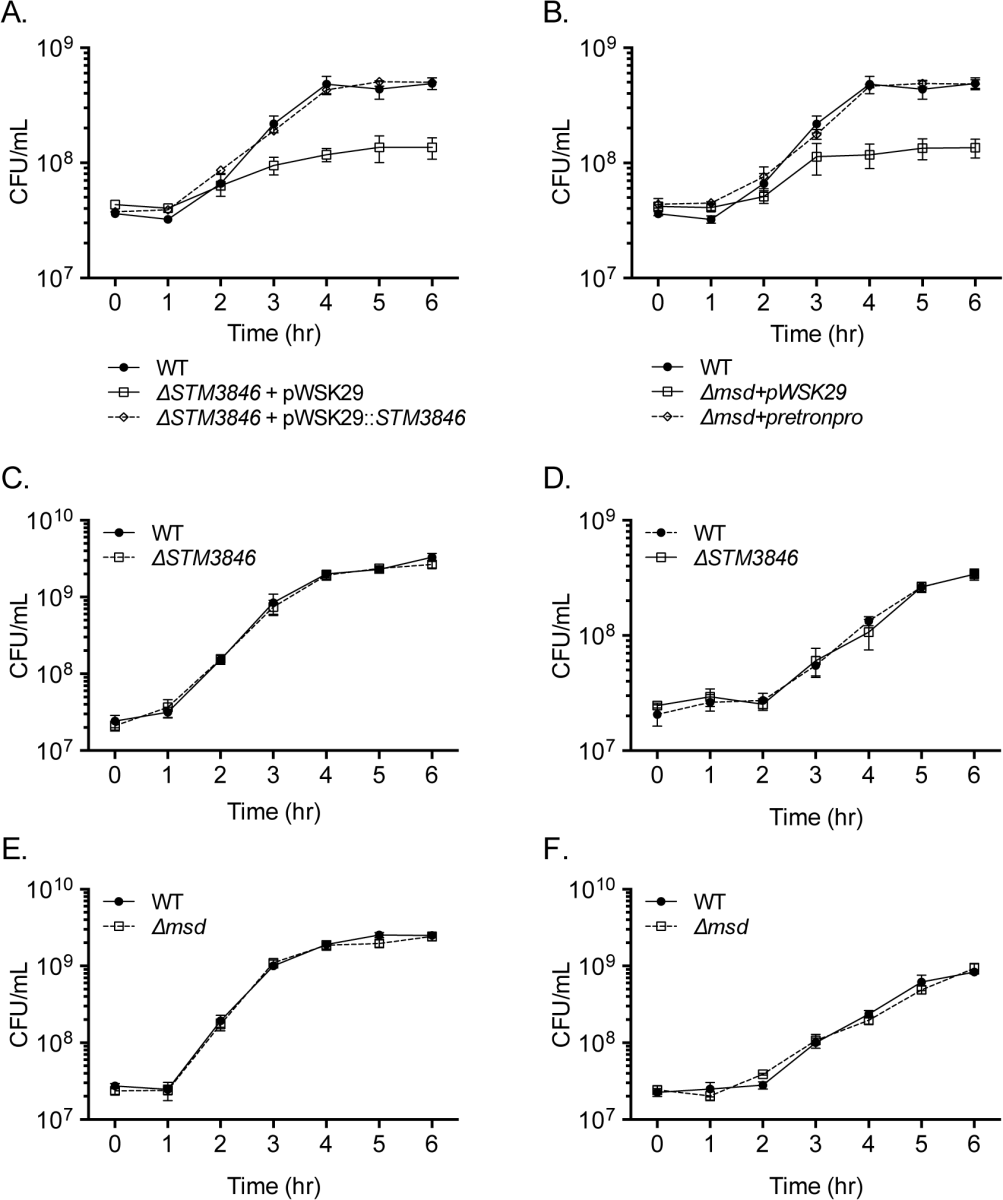
Mutants lacking msDNA are defective for anaerobic growth. (A-B) Overnight cultures of WT (closed circles; HA420), Δ*STM3846* (A; JE122) or Δ*msd* (B; JE144) with empty vector (open squares), and complemented Δ*STM3846* (A; HA1446) or complemented Δ*msd* (B; JE145) mutant (open diamonds) were transferred into an anaerobic chamber, sub-cultured 1:100 into LB broth pre-incubated for 24 hours in anaerobic conditions, and incubated at 37°C. CFU were determined hourly. Data shown are mean +/- SEM of 3 independent experiments. (C-D) Aerobic growth of WT (closed circles; HA420) and Δ*STM3846* mutant (open squares; HA1444) in (C) LB and (D) M9 minimal media. (E-F) Aerobic growth of WT (closed circles; HA420) and Δ*msd* mutant (open squares; JE135) in (E) LB and (F) M9 minimal media. Each data point is the mean +/- SEM of three independent experiments.

To determine whether the intestinal colonization defect of the STm msDNA mutants could be due to an inability to grow in oxygen limited conditions, we measured the growth of our mutants in the absence of oxygen, a condition where the retron is highly expressed [21]. Both mutants unable to produce msDNA have severe growth defects in rich media in anaerobic conditions (Fig 2A and 2B, S2 Fig), while the growth of these mutants in the presence of oxygen is similar to the isogenic WT in both rich and minimal media (Fig 2C–2F). The necessity for msDNA during anaerobic growth is consistent with the inability of msDNA-deficient mutants to efficiently colonize the intestine.

### msDNA as a regulator

**Fig 3:**
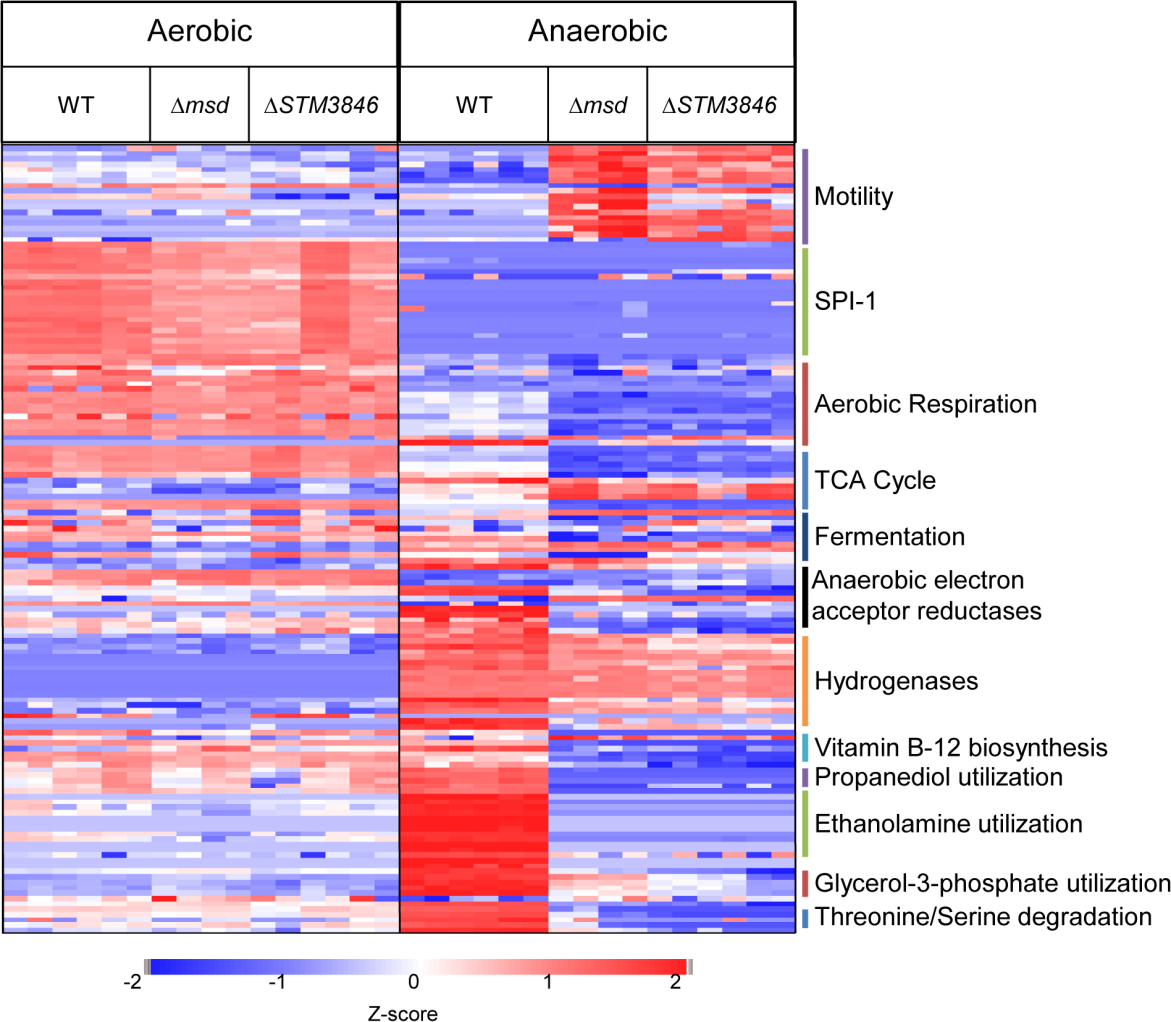
Proteins needed for anaerobic metabolism are dysregulated in mutants lacking msDNA. WT (HA420), Δ*msd* (JE135), and Δ*STM3846* (HA1444) were incubated either with agitation or statically in an anaerobic chamber for 4 hours. Proteins from bacterial cell pellets were digested and analyzed as described in Materials and Methods. Z-scores of 148 of 1504 total proteins identified from WT (n = 3) and Δ*msd* (n = 2) and Δ*STM3846* (n = 3) mutant cultures grown in both aerobic and anaerobic conditions. Functions of selected proteins are listed to the right of the heat map.

We hypothesized that msDNA might act as a *trans* regulator of gene expression for two reasons. First, small RNAs are well known to have regulatory properties through base pairing with DNA or mRNA transcripts [22]. Second, substantial over-expression of msDNA from one strain of *E*. *coli* in a heterologous strain lacking a retron resulted in small changes in the proteome [23]. To determine whether the msDNA produced by the St-85 retron might have regulatory properties, we evaluated the proteome of the WT and msDNA-deficient mutants (Δ*STM3846* and Δ*msd*) at late exponential phase, a time when the retron is expressed and msDNA is produced (Fig 1B and [24]), in both the presence and absence of oxygen. Of the 1504 total proteins identified, no significant differences in protein abundance between the WT and mutants in the presence of oxygen were detected (Fig 3 and S1 Table). This finding is consistent with previous findings that mutants lacking msDNA grow indistinguishably from the wild type organism in standard laboratory conditions (Fig 2C–2F). In addition, we noted that very few proteins differ in abundance between the Δ*STM3846* and Δ*msd* mutants, consistent with the hypothesis that the reverse transcriptase and msDNA operate in the same biological pathway.

In anaerobic conditions however, we identified 238 proteins that differed in abundance between the wild type and msDNA-deficient mutants (Fig 3 and S1 Table). Forty-three percent of proteins with reduced abundance in the mutant were involved in amino acid and carbohydrate transport/metabolism and energy production/conversion (Table 1). Twenty-five percent of all proteins of altered abundance did not belong to a functional grouping (Table 1). The abundance of proteins encoded on SPI-1 was unchanged in the absence of msDNA (Fig 3). Proteins necessary for motility were increased in abundance in anaerobically grown msDNA-deficient strains (Fig 3). However, this apparent increase did not result in a change in swimming motility of these strains in anaerobic conditions compared with the WT (S3 Fig). The abundance of numerous proteins known to be important for anaerobic growth and intestinal colonization was significantly reduced (Fig 3 and S1 Table), including proteins for 1,2 propanediol utilization [25], ethanolamine utilization [13], anaerobic sn-glycerol-3-phosphate utilization [26], anaerobic vitamin B12 biosynthesis [27], and serine/threonine degradation [28].

**Table 1:** Functional groupings of significantly dysregulated proteins in msDNA mutants. Proteins reduced in abundance, and proteins increased in abundance in msDNA mutants as determined by clusters of orthologous grouping [71].

COG grouping	# of proteins
*Proteins in reduced abundance mutants lacking msDNA relative to WT*	
amino acid transport and metabolism	32
energy production and conversion	27
carbohydrate transport and metabolism	14
cell wall/membrane/envelope biogenesis	9
coenzyme transport and metabolism	8
secondary metabolite transport, biosynthesis, catabolism	8
signal transduction	6
nucleotide transport and metabolism	5
transcription	5
inorganic ion transport and metabolism	4
post-translational modification, protein turnover, chaperone	3
intracellular trafficking, secretion, vesicular transport	2
cell cycle control, chromosome partitioning, cell division	2
cell motility	1
lipid transport and metabolism	1
general or unknown function	43
*Proteins in increased abundance in mutants lacking msDNA relative to WT*	
cell motility	8
translation ribosomal structure, and biogenesis	8
energy production and conversion	5
inorganic ion transport and metabolism	5
amino acid transport and metabolism	4
cell wall/membrane/envelope biogenesis	4
signal transduction mechanisms	3
transcription	3
coenzyme, lipid, nucleotide transport and metabolism	3
carbohydrate transport and metabolism	2
post-translational modification, protein turnover, chaperone	2
replication, recombination, and repair	2
intracellular trafficking, secretion, vesicular transport	1
general or unknown function	18

**Fig 4:**
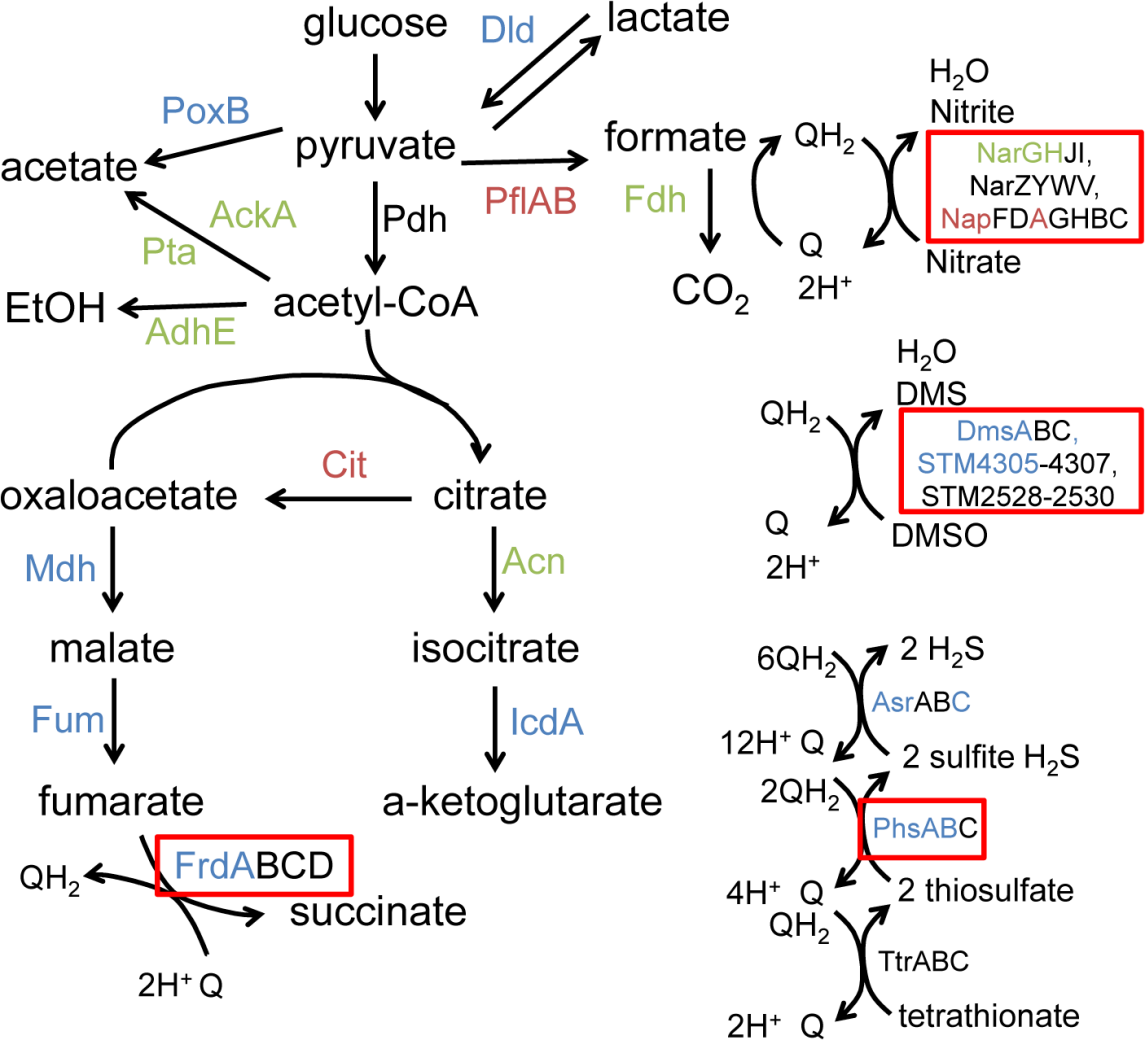
Anaerobic carbon metabolism is severely disrupted in msDNA-deficient mutants. Proteins necessary for a given reaction are listed beside the reaction. In our proteomic analysis, proteins shown in blue were present in decreased in abundance in mutants unable to produce msDNA, those shown in red are present in increased in abundance in mutants unable to produce msDNA, and those in green are unchanged in abundance in mutants unable to produce msDNA. Proteins shown in black were not observed. Boxed proteins identify alternate electron acceptors used during anaerobic conditions. Figure adapted from [30].

Numerous proteins involved in reduction of anaerobic electron acceptors [29] were altered in abundance between msDNA mutants and wild type bacteria during anaerobic growth (Fig 3). Proteins important for the reduction of thiosulfate (PhsAB) and sulfide (AsrC) were of low abundance (Fig 4 [adapted from [30]] and S1 Table). In addition, proteins necessary for the reduction of DMSO (DmsA, STM4305.s) and fumarate (FrdA) were in low abundance in mutants lacking msDNA, although they did not meet our stringent criteria for statistical significance. Expression of genes necessary to utilize alternate electron acceptors is often induced by the presence of the electron acceptor [29] so the absence of a statistically significant reduction in some of these proteins is not surprising because these compounds were not present in the growth conditions we used. Interestingly, NapA, encoding the periplasmic nitrate reductase [29], was one of the proteins that was present in increased abundance in msDNA deficient mutants compared to the WT, and there was no change in the abundance of NarGH, one of the two other nitrate reductase complexes (Fig 4 and S1 Table). These data are consistent with the growth defect of our mutants in anaerobic conditions, and suggest that msDNA-deficient mutants have a severe dysregulation of proteins necessary for reduction of terminal electron acceptors needed during anaerobiosis.

### Anaerobic terminal electron acceptor utilization in msDNA mutants

**Fig 5:**
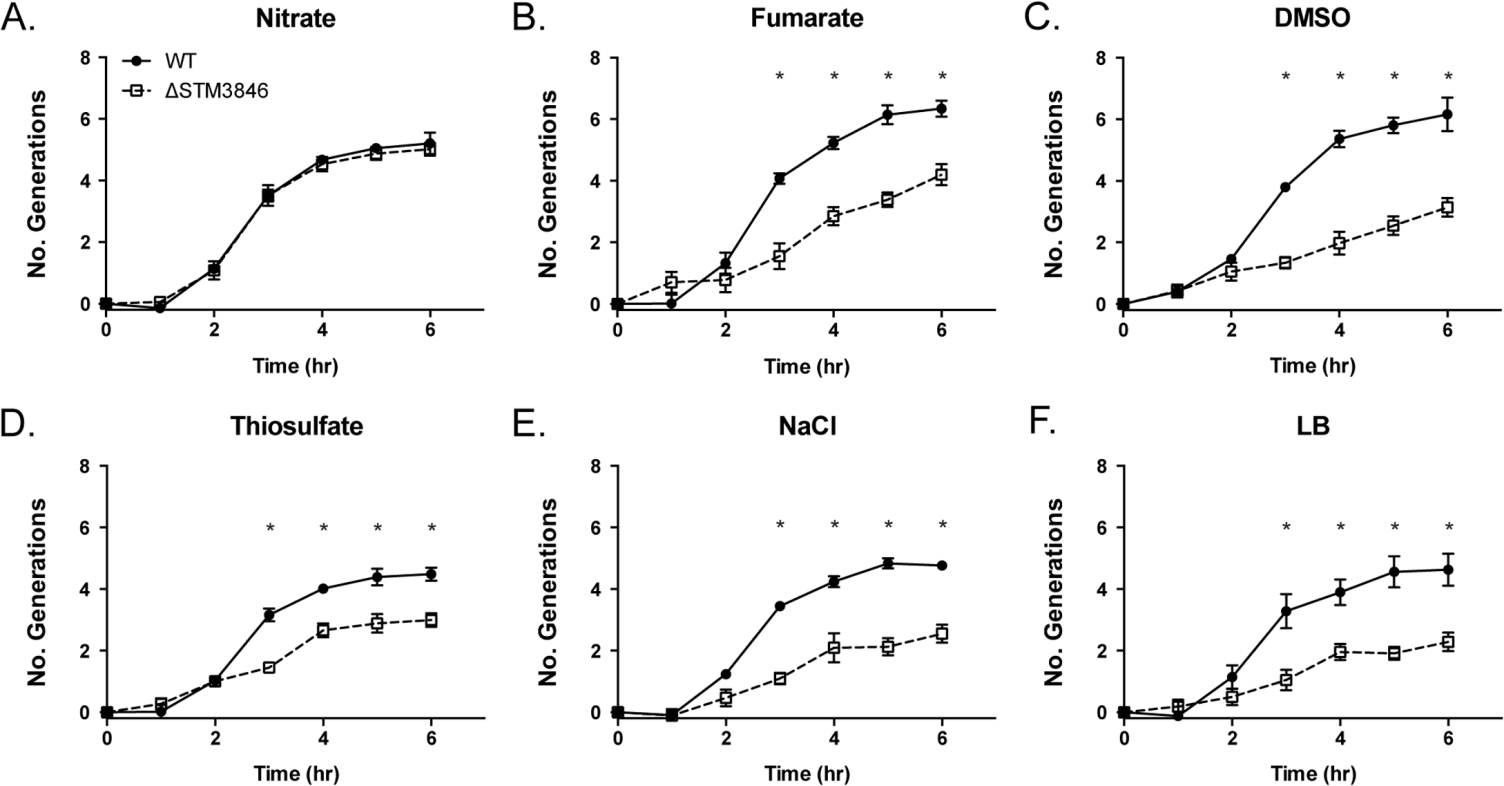
Nitrate, but not fumarate, DMSO, or thiosulfate, rescues the anaerobic growth defect of the Δ*STM3846* mutant to WT levels. WT (closed circles; HA420) and the ΔSTM3846 mutant (open squares; HA1444) were grown anaerobically as described in Fig 2, in LB with added electron acceptor: (A) Added nitrate (40 mM), (B) Added fumarate (40 mM), (C) Added DMSO (1% v/v), (D) Added thiosulfate (40 mM) or control (E) NaCl (40 mM) or (F)—LB only)]. Data points represent the mean +/- SEM of four independent experiments. Statistical significance determined by t-test on log-transformed data with * P<0.05.

**Fig 6:**
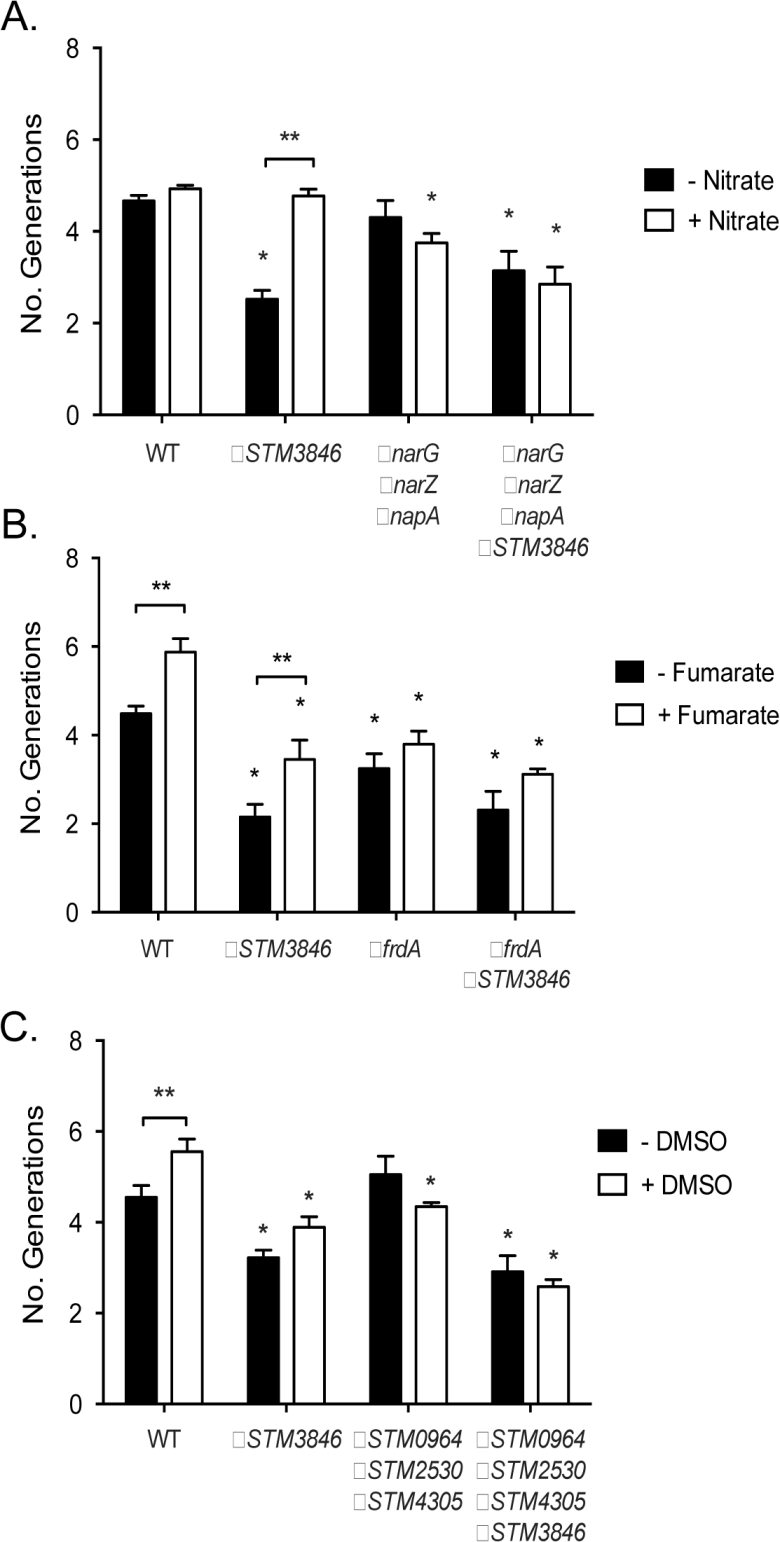
Growth defects of the Δ*STM3846* mutant in the presence of electron acceptors are linked to disruption of alternate electron acceptor reductases. Bacterial cultures were grown anaerobically for 4 hours in the presence of alternate electron acceptors as indicated in Fig 5. (A) Anaerobic growth of the WT (HA420), a Δ*STM3846* mutant (HA1444), a mutant lacking all three nitrate reductases (Δ*narG* Δ*narZ* Δ*napA;* CAL50), and a Δ*narG* Δ*narZ* Δ*napA* Δ*STM3846* quadruple mutant (JE335) in the absence (black bars) or presence (white bars) of nitrate. (B) Anaerobic growth of the WT (HA420), a Δ*STM3846* mutant (JE142), a mutant lacking the fumarate reductase (Δ*frdA;* JE309), and a Δ*frdA* Δ*STM3846* double mutant (JE334) in the absence (black bars) or presence (white bars) of fumarate. (C). Anaerobic growth of the WT (HA420), a Δ*STM3846* mutant (HA1444), a mutant lacking all three putative DMSO reductases (Δ*STM0964* Δ*STM2530* Δ*STM4305;* JE341), and a Δ*STM0964* Δ*STM2530* Δ*STM4305* Δ*STM3846* quadruple mutant (JE343) in the absence (black bars) or presence (white bars) of DMSO. Bars represent mean +/- SEM of at least three independent experiments. * Indicates significant difference between mutant and WT for that condition (P<0.05 by ANOVA). ** Indicates significant difference between conditions (P<0.05 by t-test).

Our proteomic data predict that msDNA is critical for STm to produce proteins necessary for reduction of terminal electron acceptors critical for metabolism during anaerobic conditions. In order to confirm that the reduced abundance of anaerobic terminal electron acceptor reductases, as indicated by our proteomic data, has functional consequences, we tested the ability of the addition of various terminal electron acceptors to rescue anaerobic growth of the *STM3846* mutant. We found that providing the alternate electron acceptors fumarate, DMSO, or thiosulfate to the culture media during anaerobic growth failed to restore growth of the strain lacking msDNA to WT levels (Figs 5B–5F, 6B and 6C). This finding makes sense, as our proteomic data suggest that the enzymes that transfer electrons to these terminal electron acceptors during anaerobic growth, thiosulfate reductase, sulfide reductase, fumarate reductase, and two DMSO reductases, are reduced in abundance in mutants that lack msDNA. However, the addition of nitrate to culture medium rescued the anaerobic growth of the reverse transcriptase mutant (Figs 5A and 6A). These data are consistent with our proteomic data showing that mutants lacking msDNA have adequate NarG and an increased amount of NapA allowing these strains to use nitrate as a terminal acceptor for electrons during anaerobic growth.

### msDNA and colonization of the inflamed intestine

**Fig 7:**
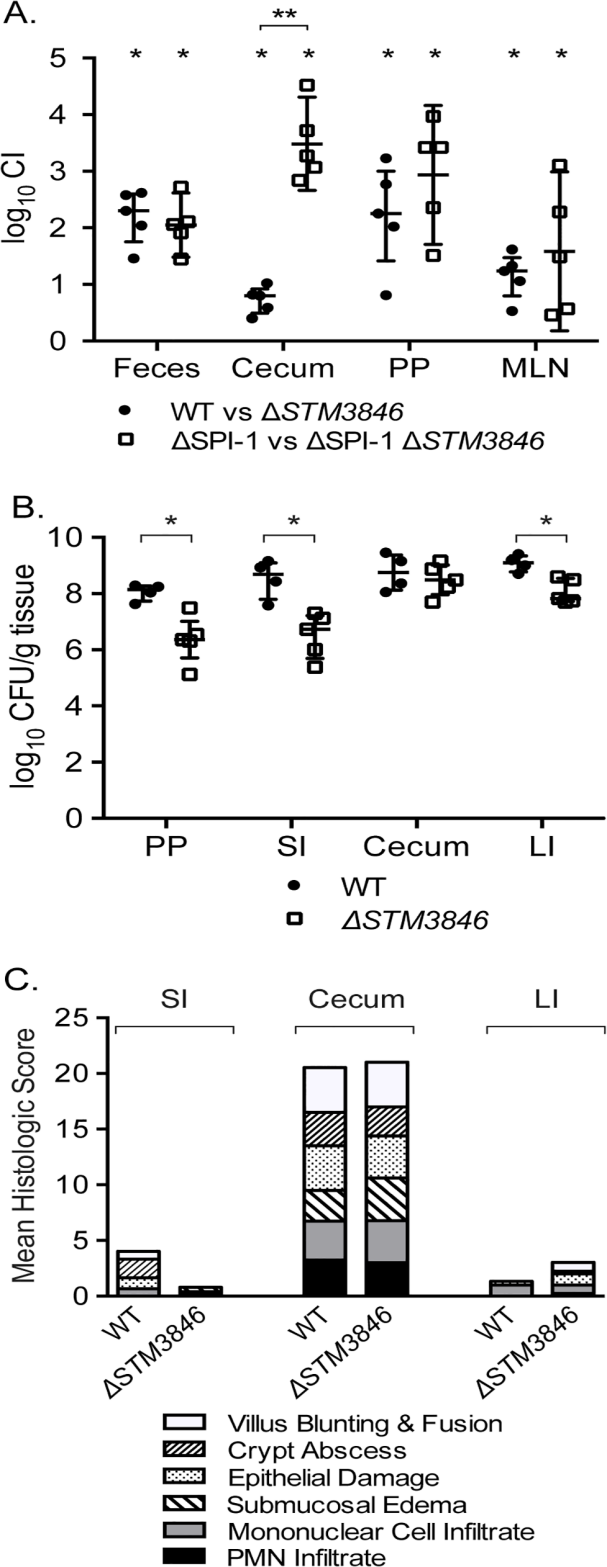
The STM3846 reverse transcriptase mediates intestinal colonization of *Salmonella* Typhimurium. (A) Competitive index of WT (JE67) vs Δ*STM3846* (JE23; n = 5 mice; closed circles) and ΔSPI-1 (HA964) vs ΔSPI-1 Δ*STM3846* (JE25; n = 5; open squares) 4 days post-infection in the murine colitis model as described in Fig 1. (B) Organ colonization of mice infected with either WT (JE67; n = 4; closed circles) or Δ*STM3846* mutant (JE23; n = 5; open squares) in the murine colitis model. (C) Mean of histologic scores from indicated intestinal segments from mice in (B). Each point (in panels A, B) represents data from a single mouse with median and interquartile range indicated. Statistical significance determined as in Fig 1. PP—Peyer’s patches; SI—small intestine; LI—large intestine.

**Fig 8:**
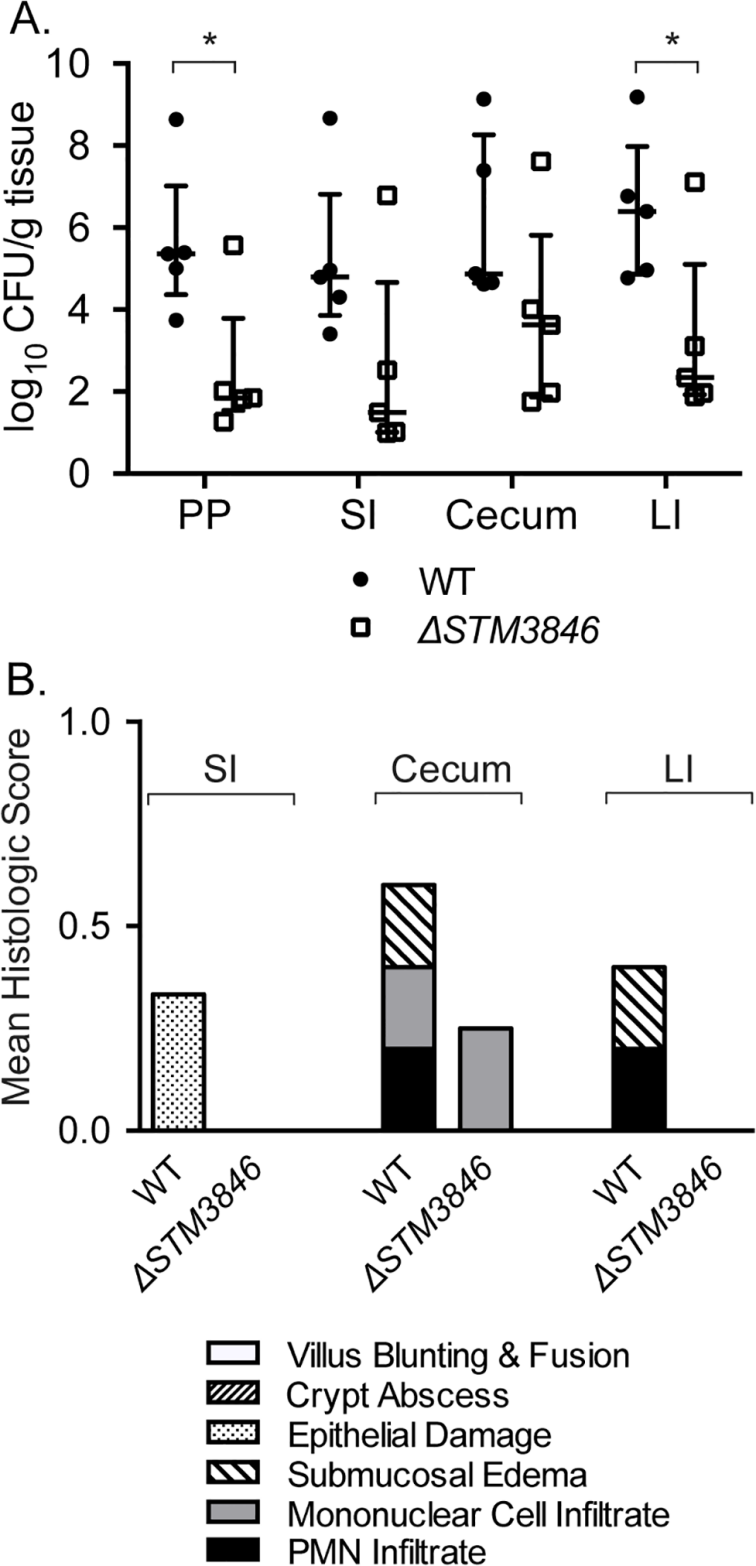
The Δ*STM3846* mutant colonizes the non-inflamed intestine poorly in the murine typhoid model. (A) Organ colonization of mice infected with either WT (JE67; closed circles; n = 5) or Δ*STM3846* mutant (JE23; open squares; n = 5). (B) Mean histologic score from mouse organs in (A). Each point represents a single mouse with median and interquartile range indicated. Statistical significance between WT and Δ*STM3846* mutant as determined by a t-test is indicated by * (P<0.05).

In the presence of an intact T3SS-1, NTS induce an inflammatory response that includes recruitment of luminal neutrophils and induction of inducible nitric oxide synthase as part of the inflammatory response [9,10,31], resulting in generation of tetrathionate and nitrate as available terminal electron acceptors in the inflamed intestine. To determine whether the colonization defects we observed were dependent on a functional T3SS-1 and host neutrophilic inflammatory response, we performed competitive infection experiments between the virulent WT and the Δ*STM3846* mutant both in the presence and absence of SPI-1 (Fig 7A). We observed that a Δ*STM3846* mutant colonizes the intestine poorly and associated organs. The modest colonization defect may be due to an inability to utilize carbon and amino acid sources within the inflamed intestine [13], or due to poor growth compared with WT prior to the host inflammatory response. Interestingly, the colonization defect of the Δ*STM3846* mutant in the mouse cecum was exacerbated in the absence of a functional T3SS-1, suggesting that a robust inflammatory response partially rescues mutants unable to produce msDNA (Fig 7A). Consistent with this finding both the small and large intestines, which lack appreciable neutrophilic inflammation (Fig 7C), are poorly colonized with the Δ*STM3846* mutant in mice inoculated with this strain alone (Fig 7B). In murine models that do not develop a neutrophilic infiltrate in the intestine in response to infection (murine typhoid model), the Δ*STM3846* mutant also colonizes poorly after oral infection (Fig 8A and 8B). Our results suggest that *STM3846* is essential for STm to colonize the intestine, a defect that is partially rescued in the presence of a profound host inflammatory response, supporting the necessity for intact anaerobic metabolic pathways in intestinal colonization.

### msDNA in enteropathogenic *E*. *coli*

**Fig 9:**
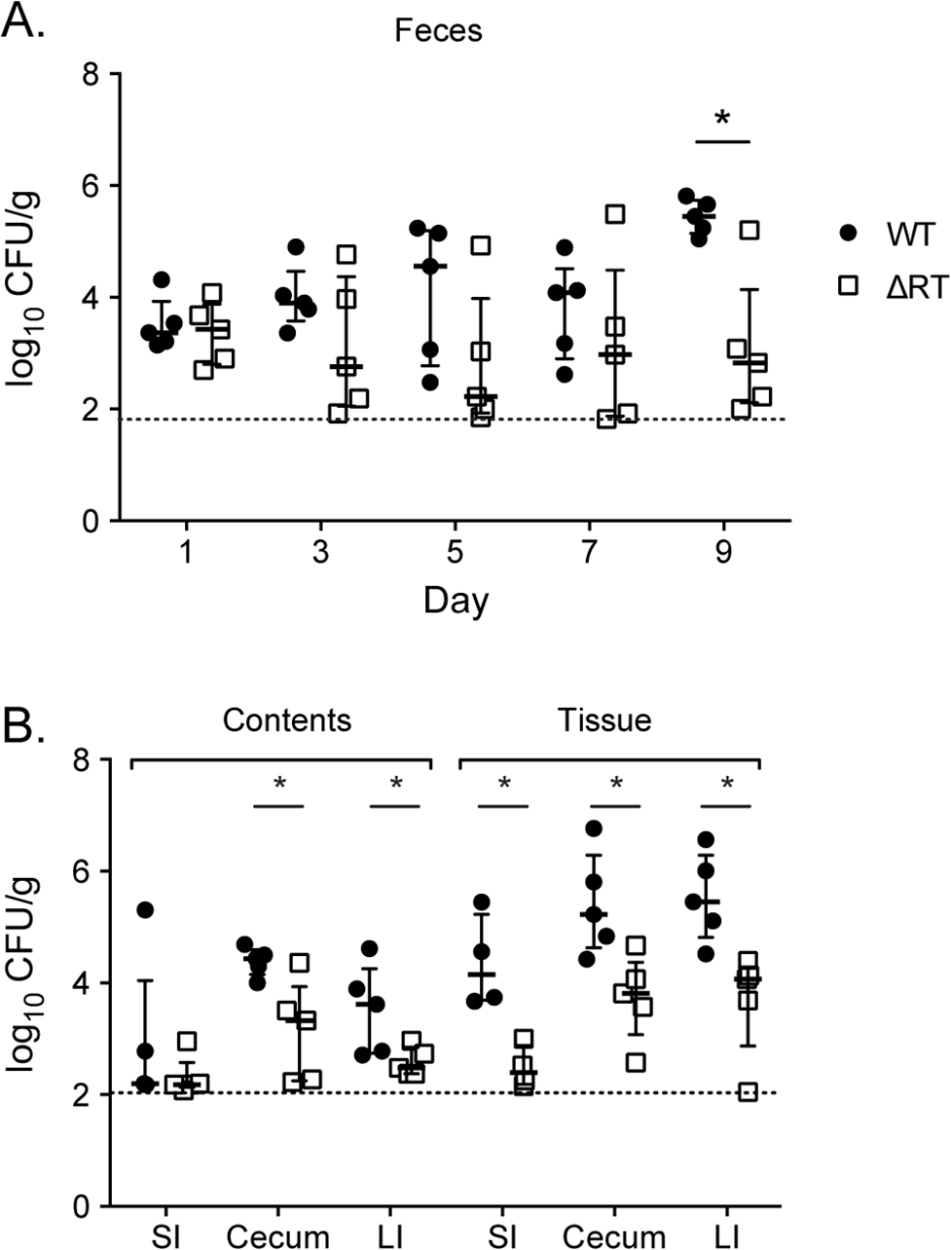
Enteropathogenic *E*. *coli* is dependent on the retron reverse transcriptase to persist within the murine intestine. Five C57Bl/6 mice were infected with an equivalent mixture of EPEC O127:H6 (JE301) or the ΔRT mutant (JE304; Δ*E2348C_3890*) by gavage. Feces were collected every two days and mice euthanized at day 10 post-infection. Intestinal contents were removed and intestinal tissue washed in PBS then contents and tissue were homogenized, diluted, and plated separately. Organs were weighed and the abundance of WT and mutant determined per gram of tissue. (A) Fecal shedding of WT (closed circles) and ΔRT mutant (open squares) throughout infection and (B) in intestinal contents (luminal bacteria) and washed tissue (tissue adherent bacteria) after 10 days competitive infection. Competitive index and statistical significance were determined as for Fig 1 with * P < 0.05. The horizontal line represents the lower limit of bacterial detection.

The msDNA of STm is similar in predicted secondary structure to msDNA of other enteric pathogens including enteropathogenic *E*. *coli* (EPEC; [4]), a close relative of STm. EPEC attaches to the epithelial surface causing characteristic attaching and effacing lesions and a malabsorptive diarrhea [32]. Despite the fact that the pathology caused by NTS and EPEC is distinct, both organisms colonize the intestine and cause diarrheal illness in susceptible hosts. We hypothesized that the RT of EPEC O127:H6, a serotype previously shown to produce msDNA [33], is necessary for this organism to colonize the gut. To test this hypothesis, we generated a non-polar deletion of the retron RT (*ΔE2348C_3890*) and performed competitive infections between this mutant and the WT. We found that an EPEC mutant lacking the RT fails to persist within the intestine of mice, both in the luminal contents and adherent to tissue (Fig 9). This defect was reversed by complementation *in trans* (S4 Fig). These data suggest that the importance of retron reverse transcriptases during intestinal infection is not restricted to salmonellae, and thus are likely to be more broadly applicable to enteric pathogens.

## Discussion

The natural function of msDNA has remained elusive despite more than 30 years of study [1,2,5,34–38]. We describe the first phenotypes for any mutant lacking msDNA. Using the enteric pathogen *S*. Typhimurium, we show that msDNA produced by a retron reverse transcriptase is critical for efficient colonization of the mammalian intestine. In STm, msDNA is critical for the ability to grow in the absence of oxygen. Identification of these phenotypes creates the first opportunity for detailed studies of the molecular function of msDNA since the discovery of these unique molecules. We further showed that STm msDNA directs colonization of the intestine through regulation of the abundance of proteins necessary for central anaerobic metabolism. Thus, our data suggest that the natural function of msDNA may be to control protein abundance, the first natural function to be ascribed to any msDNA molecule.

In STm, msDNA is produced by the STM3846 reverse transcriptase using *msd* as a template sequence and *msr* as a primer. The msDNA from STm has a predicted 85-nucleotide DNA stem with no mismatched base pairs and a 4-nucleotide loop, and an RNA portion with two predicted smaller imperfect stem loop structures [4]. The RNA and DNA portions of msDNA are covalently joined by a unique 2’5’ phosphodiester linkage on a conserved guanine [39]. It is unclear whether the entire *msr* RNA sequence remains in the mature STm msDNA. Consistent with prior reports [34,39], we showed that both the RT and *msd* are requirements for production of msDNA. The intervening ORF, *STM3845*, is dispensable for msDNA production. This is perhaps not surprising as the presence of another ORF in addition to the RT in retrons is relatively rare, and appears to be more common on retrons borne by pathogens [4,40,41]. It has been suggested that the retron RT could produce a variety of different cDNA molecules if the sequence of the mRNA transcript is identical to that of the 5’ end of *msr* [42]. However, we observed similar defects in intestinal colonization and anaerobic growth of mutants lacking either the RT or *msd*. These data suggest that it is msDNA, and not some other potential product of the RT, that mediates intestinal colonization of STm.

Previous attempts to evaluate the function of msDNA have used artificial systems, failing to identify phenotypes for mutants lacking msDNA and to definitively identify the natural function of these molecules [23,43–47]. When an msDNA from one strain of *E*. *coli* with mismatched base pairs in the predicted DNA stem region is significantly overexpressed in a heterologous strain of *E*. *coli* lacking its own retron, the frequency of spontaneous mutation was increased due to sequestration of mismatch repair proteins [43,44,46]. Thus, the production of msDNA was thought to increase mutation frequency. However, no previous work has demonstrated that deletion of msDNA from a bacterium naturally producing msDNA decreases mutation frequency. We hypothesize that substantial over-expression of any mismatched DNA could increase mutation frequency by the same mechanism. Thus, this previous finding may not illuminate the true function of msDNA in the cell.

When mutants lacking the ability to make msDNA are grown without oxygen, 15% of all proteins we could identify were in altered abundance. However, no dysregulated proteins were identified during aerobic growth, consistent with the lack of identifiable phenotypes in the presence of oxygen. Our proteomic data were generated using cultures grown for the same duration of time under varying growth conditions. Some of the differences in protein abundance may result because wild type and mutant that cannot make msDNA grow differently during anaerobic conditions. However, our growth data suggest that the growth phase of the wild type and mutants unable to make msDNA are not dramatically different at the times we chose to collect samples for our analysis. Furthermore, the differences in protein abundance between the msDNA mutant and the wild type during anaerobic growth that we re-tested appear to be functionally significant. We show that the growth of mutants that cannot make msDNA, and that have reduced abundance of several alternate electron acceptor reductases needed during anaerobic growth, cannot be rescued by addition of the cognate alternate electrons. Furthermore, the msDNA mutant overproduces periplasmic nitrate reductase (NapA) and a wild type level of a second nitrate reductase (NarG). We show that these proteins and thus this pathway are functional, as the exogenous addition of the terminal electron acceptor nitrate rescues the anaerobic growth of mutants unable to make msDNA. Prior reports suggest that the DNA portion of msDNA can be engineered to act as a regulatory molecule by creating an antisense sequence in the DNA loop [45]. Our data suggest that the natural function of msDNA may be to act as a regulatory molecule although we have not yet identified specific regulatory targets.

There are two known master regulators of anaerobic metabolism in facultative anaerobes: *fnr* and *arcA* [48]. The transcriptional and protein profiles of anaerobically-grown *Salmonella* mutants deficient in *fnr* and *arcA* are established [49,50]. With few exceptions, the proteins of altered abundance in our proteomic data align poorly with genes regulated by either *fnr* or *arcA*. However, it is difficult to draw meaningful comparisons across our proteomic data and published transcriptional profiles of mutants grown in the absence of oxygen, because of protein profiles with transcript abundance are not directly comparable. Our data raise the possibility that regulation by msDNA may represent an additional pathway to regulate the abundance of proteins necessary for anaerobic metabolism. Further mechanistic study of the anaerobic regulation of gene and protein expression is critical to understanding the behavior of *Salmonella* in intestinal colonization.

Recent evidence suggests that *Salmonella* exploits the host inflammatory response to gain a competitive advantage in the intestinal lumen [10–13]. Reactive oxygen species produced by neutrophils oxidize thiosulfate to tetrathionate, a compound that *Salmonella*, but not resident microflora, uses as a terminal electron acceptor [10]. Epithelial-derived nitrate also contributes to the growth of *Salmonella* in the anaerobic conditions of the intestine by acting as a preferred electron acceptor in these conditions [31]. Some nutrients, such as ethanolamine, are used only during the neutrophilic inflammatory response [13]. We have shown that some of these processes in STm are altered in mutants unable to produce msDNA, along with many other proteins with less clearly defined roles in pathogenesis.

We also observed a defect in intestinal persistence of an EPEC mutant lacking its retron RT. *Salmonella enterica* and *E*. *coli* are close phylogenetic relatives and both cause diarrheal illness in susceptible hosts, but there are critical differences in the retron between organisms. The RT of EPEC O127:H6 is located in a different genomic context than the retron of STm and has a GC content of 51.8%, similar to the average GC content of 50.6% [51] suggesting that this gene was not acquired recently. This GC content in the EPEC retron is in contrast to the GC content of the retron of STm, 30.6% compared with the average GC content of 52.4% [4,52]. Unlike STm, the retron of EPEC O127:H6 lacks an additional ORF. The predicted secondary structures of msDNA from EPEC and STm are similar, however EPEC msDNA is predicted to have mismatched base pairs in the DNA stem [4]. Despite these differences, we report that EPEC mutants lacking the retron RT also have a phenotype during colonization of the intestine.

Critical differences also exist between the pathogenesis of EPEC and STm diarrheal diseases. In the intestine, *Salmonella* lives both in the lumen and invades the epithelium, replicating intracellularly and inducing a profound neutrophilic inflammatory diarrhea [53]. In contrast, EPEC attaches to the intestinal epithelium below the intestinal mucus in these regions of the gastrointestinal tract and remains extracellular [54]. Although our understanding of the molecular mechanism of the development of diarrhea during EPEC infection is incomplete, this infection causes a secretory diarrhea [32]. Thus, the mechanism of EPEC-induced diarrhea is substantially different than the inflammatory diarrhea caused by non-typhoidal salmonellae. Our data suggest that retron RTs are critical for colonization of the intestine by both of these pathogens, yet the phenotypes of these mutants in *Salmonella* versus EPEC during infection are different. While the role of retron reverse transcriptases and msDNA in intestinal colonization by enteric pathogens is likely to be ubiquitous, we hypothesize based both on our data and on the differences in diseases between these two organisms, that the processes regulated and the regulatory targets themselves are likely to be different.

We show that a natural function of msDNA is to regulate protein abundance, the first reported natural function of any msDNA molecule. STm mutants unable to make msDNA poorly colonize the murine intestine. This colonization defect is due to altered abundance of numerous proteins, including those necessary for central anaerobic metabolism, a process known to be necessary for the ability of STm to colonize the intestine of mammals. We observed that an EPEC mutant lacking its retron reverse transcriptase has a reduced ability to persist in the murine intestine, suggesting that the presence and function of msDNA may be broadly applicable to other enteric pathogens. Retrons are also widespread in non-pathogenic eubacteria (Reviewed in [3]) including most isolates of the environmental bacterium *Myxococcus xanthus* [35]. msDNA is present in high copy per cell [1], suggesting that the regulatory function of this molecule is critical for the lifestyle of the host bacterium. It is puzzling that this molecule appears to have a function under only certain conditions despite the fact that it is produced in abundance. One possible explanation for this phenomenon is that msDNA may sense environmental changes in order to regulate gene expression. This hybrid RNA-DNA molecule represents an exciting new class of bacterial regulatory molecules with broad application to the understanding of the lifestyles of pathogens and non-pathogens alike.

## Materials and Methods

### Bacterial strains

All bacterial strains, plasmids, and primers used for mutant construction are listed in S2 Table, S3 Table). All *Salmonella* strains are derivatives of ATCC 14028s. Enteropathogenic *E*. *coli* O127:H6 strain E2348/69 [55], a generous gift of M. Donnenberg, is the genetic background for all EPEC mutants described here. Mutants were constructed using a modification of the lambda-red recombination technique and antibiotic resistance cassettes removed as previously described [56,57] [58]. All *Salmonella* mutations were moved into a clean genetic background by P22 transduction [59]. Standard cloning protocols were used to generate complementing plasmids [60].

All bacterial cultures were grown at 37°C aerobically with vigorous agitation or standing in an anaerobic chamber with internal atmosphere of 5% H_2_, 5% CO_2_, and 90% N_2_ (Bactron I, ShelLab). For anaerobic growth experiments, bacteria were grown overnight aerobically then transferred into the anaerobic chamber and diluted 1:100 into media pre-equilibrated for at least 18 hours. Alternate electron acceptors (Sigma-Aldrich) sodium nitrate, sodium fumarate, sodium thiosulfate, and sodium tetrathionate were added to LB to a final concentration of 40mM. Sodium chloride (Sigma-Aldrich) at a final concentration of 40 mM served as a negative control. DMSO (Sigma-Aldrich) was added to LB to a final concentration of 0.1% (v/v). Bacteria were grown in Luria-Bertani (LB) broth or LB or MacConkey (Difco) agar supplemented with the following antibiotics as appropriate: kanamycin (50 mg/L), nalidixic acid (50 mg/L), carbenicillin (100 mg/L), streptomycin (100 mg/L), and chloramphenicol (20 mg/L).

All experiments were performed on at least three separate occasions. Bacterial generation number was calculated using the following equation: [log_10_(CFU final)—log_10_(CFU start)]/log_10_(2).

### Mouse infections

Ethics Statement: This study was performed in strict accordance with the recommendations in the Guide for the Care and Use of Laboratory Animals of the National Institutes of Health. The Institutional Animal Care and Use Committees of Texas A&M University and North Carolina State University approved all animal experiments (protocol numbers 2012–084 and 2011–167 (TAMU) and 14–132-B (NCSU)). All experiments that utilized mice were performed using 8–12 week old female C57BL/6J mice (Jackson Laboratories). For competitive infection experiments, mice were infected by gavage with an equivalent ratio of WT and mutant bacteria. The competitive index was determined by dividing the ratio of WT to mutant bacteria in the selected organ by that ratio in the inoculum. For single infections, mice were infected with either WT or mutant bacteria. The harvested tissue was weighed, homogenized, and CFU was determined per gram of tissue collected.

*Salmonella* infections were performed as previously described [15]. For the murine colitis model, mice were administered 20 mg streptomycin in 75 μL sterile water by gavage. Twenty-four hours after treatment, mice were infected with approximately 10^8^ CFU of *Salmonella* in 100 μL volume by gavage. Feces were collected 24 hours after infection. Mice were euthanized by carbon dioxide asphyxiation at 96 hours post-infection and organs harvested, homogenized, serially diluted, and plated on LB agar with appropriate antibiotics for enumeration of CFU. For the murine typhoid model, mice were treated with 75 μL sterile water by gavage. Mice were then infected and euthanized as above.

EPEC mouse infections were performed essentially as previously described [61]. Mice were infected with approximately 10^8^ CFU in 100 μL volume by gavage. Feces were collected every other day for 9 days. Mice were euthanized 10 days post-infection. The aboral 5 cm of small intestine, the entire cecum, and the entire colon were collected. Intestinal contents were exposed through a longitudinal incision. The intestinal segment was placed into sterile PBS and vigorously agitated to remove intestinal contents. Intestinal tissue was washed in sterile PBS to remove remaining ingesta. Intestinal contents and tissue were homogenized separately, serially diluted, and plated on MacConkey agar and LB agar with appropriate antibiotics to enumerate CFU.

### Histopathology

Samples from mouse ileum, cecum, and transverse colon were collected 96 hours post-infection and fixed in formalin. All tissues were routinely processed and stained with hematoxylin and eosin. All histologic analyses were performed by a veterinary pathologist blinded as to infection group. Tissues were scored (0–4) for each of the following parameters: polymorphonuclear cell (PMN) infiltration, mononuclear leukocyte infiltration, crypt abscess, submucosal edema, villus blunting, and epithelial damage as described [13,15,62,63].

### msDNA isolation

msDNA was isolated from aerobic late log phase cultures normalized by OD_600_. Bacteria were lysed as for plasmid isolation (Qiagen Mini-prep) and msDNA isolated from the filtered fraction with subsequent ethanol precipitation. msDNA was visualized using a native polyacrylamide gel with in-gel ethidium bromide staining.

### Invasion assays

Cell lines were purchased from American Type Culture Collection (ATCC) and used within 15 passages. HeLa cells (human cervical adenocarcinoma epithelial, ATCC CCL-2) were grown as recommended by ATCC. HeLa cells were seeded in 24-well plates at 5 x 10^4^ cells/well approximately 24 h prior to infection.

Late-log phase cultures were prepared by inoculating 10 ml LB broth with 0.3 ml overnight shaking culture. Flasks were grown at 37°C with agitation for 3 hours. Bacteria were collected by centrifugation at 8000 x g for 90 seconds, resuspended in an equal volume of Hanks’ buffered saline solution (HBSS, Mediatech) and added directly to mammalian cells seeded in 24-well plates for 10 minutes. The multiplicity of infection was approximately 50. Non-internalized bacteria were removed by aspiration. Monolayers were washed three times in HBSS and were then incubated in growth media until 30 min post-infection. Thereafter, gentamicin was added at 50 μg/ml from 30–90 min p.i. to kill extracellular bacteria and reduced to 10 μg/ml from 90 min post-infection For enumeration of intracellular bacteria, monolayers were washed once in phosphate-buffered saline, and then solubilized in 0.2% sodium deoxycholate and serial dilutions were plated on LB agar.

### Motility assays

Swimming motility was performed as previously described [64]. Swimming was assayed on plates containing 0.3% Difco Bacto Agar (LB agar base 25g/L). Plates were incubated either in open air or in the anaerobic chamber overnight prior to use for swimming assays. Overnight cultures of bacterial strains were grown at 37°C with agitation and cell numbers normalized by optical density. An aliquot of each normalized culture was transferred into the anaerobic chamber. The WT, Δ*STM3846*, and Δ*msd* mutants (3 μl each) were spotted onto the same swimming agar plate and incubated at 37°C aerobically or anaerobically for 5 hours. The diameter of the cell spread was measured and compared with that of the WT on the same plate. Each assay was performed in triplicate on three independent occasions (anaerobic) or in four replicates on two independent occasions (aerobic).

### Data analysis

Statistical analysis was performed using GraphPad Prism 6. All data were log transformed prior to analysis. Statistical significance was set at P < 0.05 and was determined using a t-test or ANOVA where indicated.

### Proteomic analysis

Aerobic overnight cultures of the wild type and the Δ*STM3846* and Δ*msd* mutants were diluted 1:100 and incubated either aerobically or in an anaerobic chamber (Coy) for 4 hours on three independent occasions. Bacteria were pelleted and supernatants discarded. Cell pellets were resuspended in 100 mM NH_4_HCO_3_, pH 8.0 and lysed by vigorous vortexing in the presence of 0.1 mm silica/zirconia beads. Proteins were denatured and reduced with 8M urea and 5 mM dithiothrietol, respectively, for 30 minutes at 60°C. The proteins underwent enzymatic digestion for 3 hours at 37°C with 1/50 enzyme/protein (w/w) ratio of sequencing-grade trypsin. The resultant peptides were desalted for mass spectrometric (MS) analysis using C18 solid phase extraction cartridges (50 mg, 1 mL, Discovery, Supelco). The cartridges were activated with methanol, followed by equilibration with 0.1% TFA before loading the samples. The cartridges were then washed with 5% acetonitrile (ACN)/0.1% TFA and eluted with 80% ACN/0.1% TFA. Eluted peptides were concentrated in the vacuum centrifuge and diluted to a concentration of 0.5 mg/mL with water for the MS analysis.

Digested peptides were loaded into capillary columns (75 μm x 35 cm, Polymicro) packed with C18 beads (3 μm particles, Phenomenex) connected to a custom-made 4-column LC system [65]. The elution was performed using the following gradient: equilibration in 5% B solvent, 5–8% B over 2 min, 8–12% B over 18 min, 12–35% B over 50 min, 35–60% min over 27 min and 60–95% B over 3 min. (solvent A: 0.1% FA; solvent B: 90% ACN/0.1% FA) and flow rate of 300 nL/min. Eluting peptides were directly analyzed either on an Orbitrap (LTQ Orbitrap Velos, Thermo Scientific, San Jose, CA) mass spectrometer using chemically etched nanospray emitters [66]. Full scan mass spectra were collected at 400–2000 m/z range and the ten most intense ions were submitted to low-resolution CID fragmentation once (35% normalized collision energy), before being dynamically excluded for 60 seconds.

Tandem mass spectra were searched with MSFG+ against *Salmonella enterica* serovar Typhimurium 14028s and common contaminant sequences (downloaded from NCBI, all in forward and reversed orientations), using the following parameters: (i) partial tryptic digestion, (ii) 50 ppm parent mass tolerance, (iii) methionine oxidation as a variable modification. The peptides were filtered with a MSGF probability score [67] ≤ 1x10^–9^. Peak areas for each peptide were retrieved using the MultiAlign tool [68], and to ensure the quality of peptide-to-peak matching, the data was filtered with a Statistical Tools for AMT tag Confidence (STAC) score ≥ 0.7 and uniqueness probability ≥ 0.5 [69]. Additionally, proteins were required to have at least 2 peptides and at least one peptide with STAC ≥ 0.9. Peptide abundance values were log transformed and rolled-up into proteins using Qrollup tool, available in DAnTE [70]. Abundance values for each protein across all 32 conditions (WT, mutants, anaerobic, aerobic conditions, biological replicates, and technical replicates) were used to calculate a Z-score for each measurement where missing values were filled with 19.5. The Z-score transformation enables comparisons of trends across conditions and proteins to identify relevant abundance changes.

## Supporting Information

S1 Fig The Δ*msd* mutant has a mild invasion defect in cultured epithelial cells. (A) Invasion efficiency of Δ*STM3846* (HA1444) and Δ*msd* (JE135) mutants into HeLa cell monolayers normalized to the efficiency of the WT (HA420) at 1 hour post-infection. (B) Fold-replication of the Δ*STM3846* and Δ*msd* mutants 7 hours post-infection/1 hour post-infection normalized to fold-replication of the WT. Error bars represent the mean +/- SD. Invasion was measured on five separate occasions, and intracellular replication on three separate occasions. * P<0.05.
(TIF)

S2 Fig Anaerobic growth curves of Δ*STM3846* and Δ*msd* mutants. Anaerobic growth curves were performed as described (Fig 2) using mutants lacking plasmids (HA1444 and JE135).
(TIF)

S3 Fig Motility of msDNA-deficient mutants does not depend on the presence of oxygen. Normalized overnight cultures of WT (HA420), Δ*STM3846* (HA1444), and Δ*msd* (JE135) were spotted onto swimming agar either in the presence of oxygen or in an anaerobic chamber. Cell spread was measured 5 hours post-inoculation and compared with that of the WT growing on the same plate. Bars represent the mean +/- SD. Anaerobic swimming was measured in triplicate on three separate occasions and aerobic swimming measured in quadruplicate on two separate occasions. (*) significant difference between WT and the mutant. (**) significant difference between mutants. P<0.05.
(TIF)

S4 Fig The persistence defect of the EPEC ΔRT mutant is reversed by complementation *in trans*. Two groups of five C57BL/6 female 8–12 week old mice were infected with 10^8^ CFU of an equivalent mixture of EPEC O126:H7 (JE301) and ΔRT mutant (Δ*E2348C_3890*) bearing the empty plasmid (JE472; closed circles) or complementing plasmid (JE470; open boxes). Mice were euthanized 10 days post-infection and organs harvested to determine CFU. Data presented are the composite of two independent experiments. Each data point represents a single animal and the median and interquartile ranges are indicated. Competitive index and statistical significance determined as described for Fig 1. * P<0.05 (WT vs mutant) and ** P< 0.05 (between infection groups.
(TIF)

S1 Table Comparison of the proteomes of wild type (HA420), *ΔSTM3846* (HA1444) and *Δmsd* (JE135) mutants during both aerobic and anaerobic growth. The mean Z-score for each bacterial strain in a given condition is shown (WT n = 6, *ΔSTM3846 mutant n = 6*, *Δmsd* mutant n = 4). SD denotes the standard deviation of Z-scores for a bacterial strain in a given condition. Cells highlighted in blue indicate decreased protein abundance relative to the wild type, while cells highlighted in red indicate increased protein abundance relative to wild type.
(XLSX)

S2 Table Bacterial strains and plasmids utilized in this study. (XLSX)

S3 Table Primers utilized to construct mutants and complementing plasmids. (XLSX)
