# Supplementary figures and images for: Multicopy Single-Stranded DNA Directs Intestinal Colonization of Enteric Pathogens
Source: PLoS Genet. 2015 Sep 14;11(9):e1005472. doi: 10.1371/journal.pgen.1005472 (PMC4569332; doi:10.1371/journal.pgen.1005472)

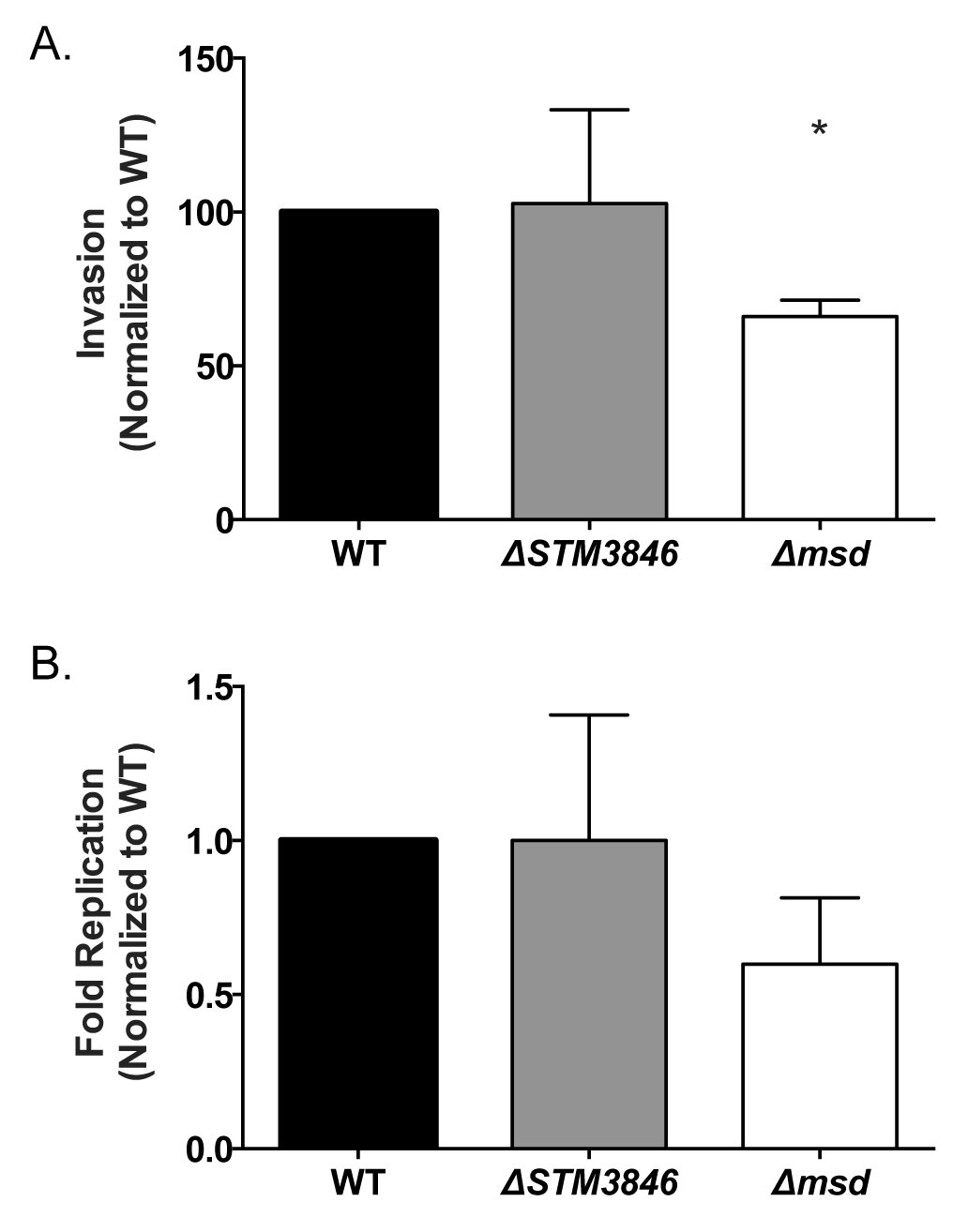

Supplement: S1 Fig — (A) Invasion efficiency of ΔSTM3846 (HA1444) and Δmsd (JE135) mutants into HeLa cell monolayers normalized to the efficiency of the WT (HA420) at 1 hour post-infection. (B) Fold-replication of the ΔSTM3846 and Δmsd mutants 7 hours post-infection/1 hour post-infection normalized to fold-replication of the WT. Error bars represent the mean +/- SD. Invasion was measured on five separate occasions, and intracellular replication on three separate occasions. * P<0.05. (TIF) [file pgen.1005472.s001.tif]

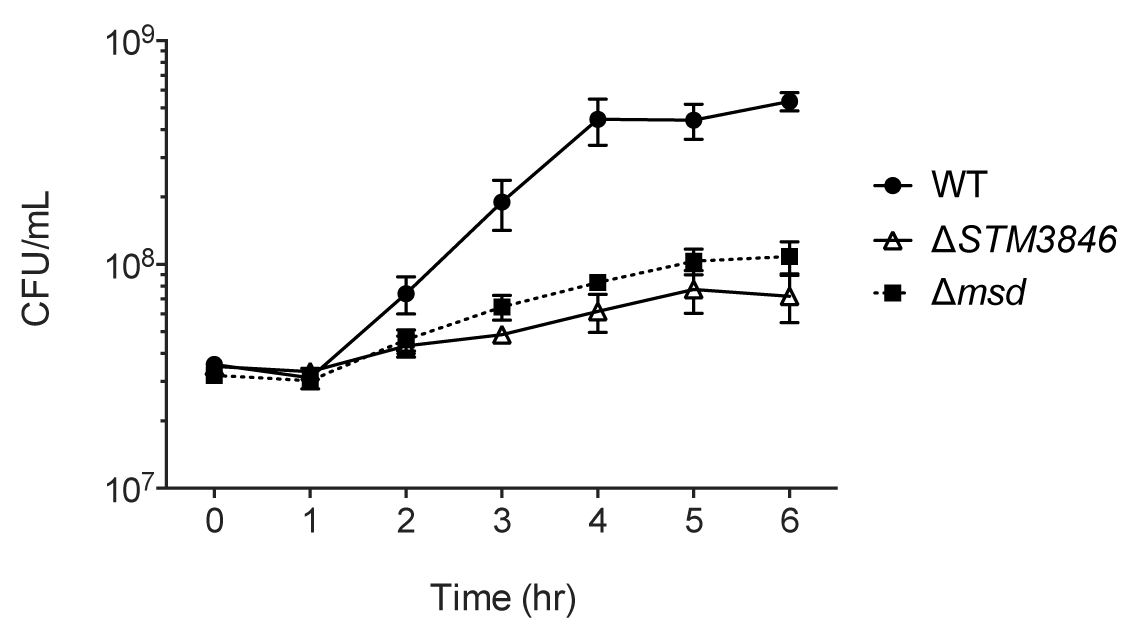

Supplement: S2 Fig — Anaerobic growth curves were performed as described (Fig 2) using mutants lacking plasmids (HA1444 and JE135). (TIF) [file pgen.1005472.s002.tif]

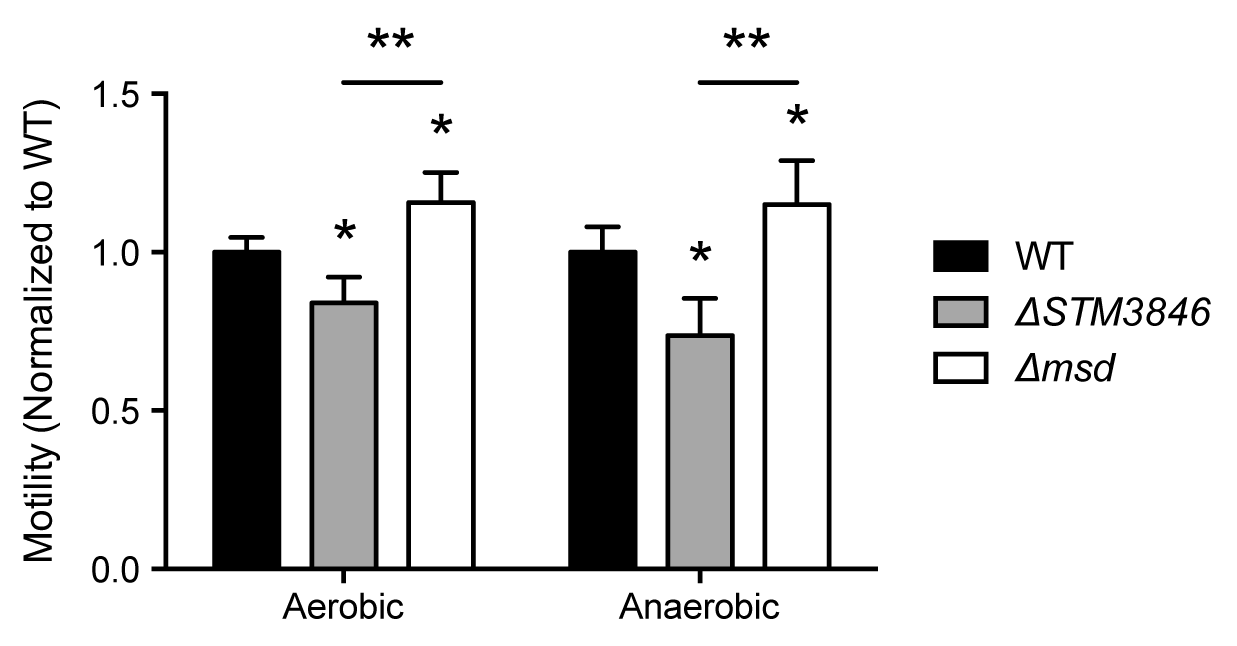

Supplement: S3 Fig — Normalized overnight cultures of WT (HA420), ΔSTM3846 (HA1444), and Δmsd (JE135) were spotted onto swimming agar either in the presence of oxygen or in an anaerobic chamber. Cell spread was measured 5 hours post-inoculation and compared with that of the WT growing on the same plate. Bars represent the mean +/- SD. Anaerobic swimming was measured in triplicate on three separate occasions and aerobic swimming measured in quadruplicate on two separate occasions. (*) significant difference between WT and the mutant. (**) significant difference between mutants. P<0.05. (TIF) [file pgen.1005472.s003.tif]

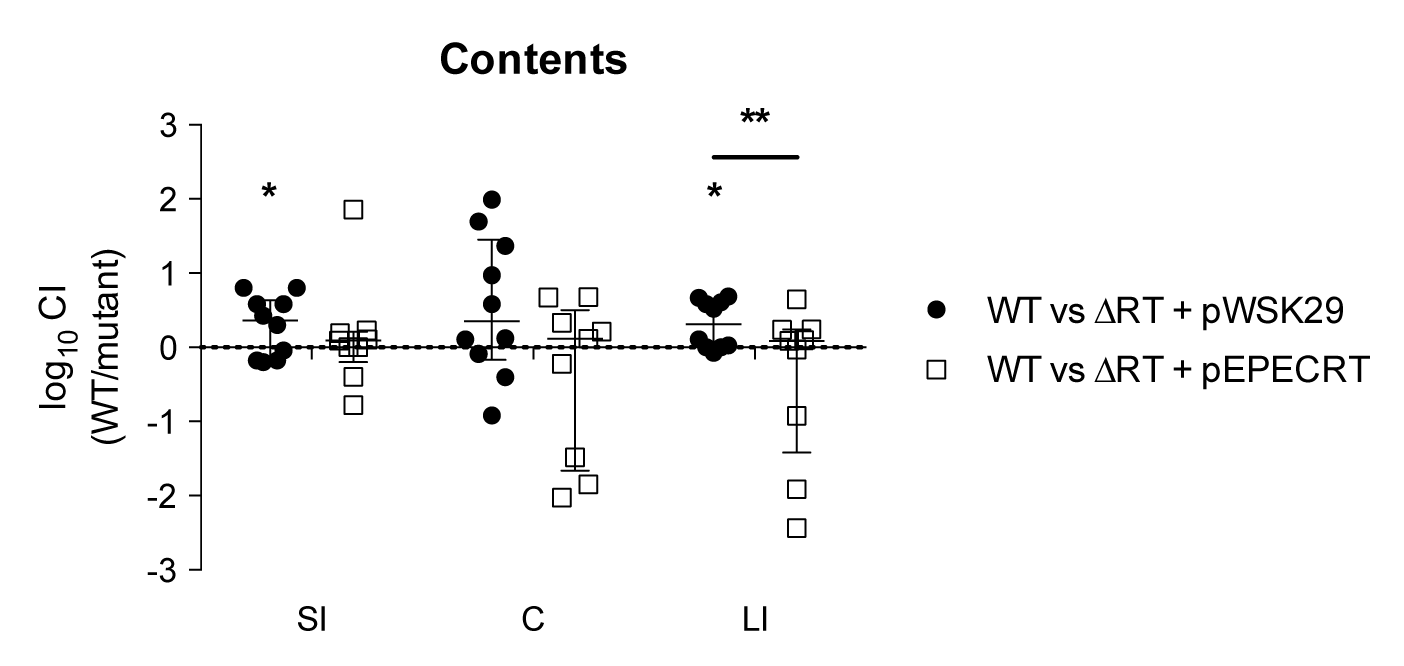

Supplement: S4 Fig — Two groups of five C57BL/6 female 8–12 week old mice were infected with 108 CFU of an equivalent mixture of EPEC O126:H7 (JE301) and ΔRT mutant (ΔE2348C_3890) bearing the empty plasmid (JE472; closed circles) or complementing plasmid (JE470; open boxes). Mice were euthanized 10 days post-infection and organs harvested to determine CFU. Data presented are the composite of two independent experiments. Each data point represents a single animal and the median and interquartile ranges are indicated. Competitive index and statistical significance determined as described for Fig 1. * P<0.05 (WT vs mutant) and ** P< 0.05 (between infection groups. (TIF) [file pgen.1005472.s004.tif]
